# Afforestation suppresses *Oncomelania hupensis* snail density through influencing algae in beaches of the Dongting Lake

**DOI:** 10.1371/journal.pntd.0009100

**Published:** 2021-02-04

**Authors:** Xiao Yang, Qian Zhang, Li Ma, Qi-Xiang Sun, Song Liang, Jin-Xing Zhou

**Affiliations:** 1 School of Soil and Water Conservation, Beijing Forestry University, Beijing, China; 2 Academy of Forest Inventory and Planning, National Forestry and Grassland Administration, Beijing, China; 3 Key Laboratory of State Forestry Administration on Soil and Water Conservation, Beijing, China; 4 Engineering Research Center of Forestry Ecological Engineering, Ministry of Education, Beijing, China; 5 Institute of Forestry, Chinese Academy of Forestry, Beijing, China; 6 Department of Environmental and Global Health, College of Public Health and Health Professions, and Emerging Pathogens Institute, University of Florida, Gainesville, Florida United States of America; RTI International, UNITED STATES

## Abstract

**Background:**

Oncomelania snails serve as the sole intermediate host for Schistosoma japonicum, one of the most important neglected tropical diseases in the world. Afforestation suppression of the Oncomelania hupensis snail has been a long-term effective national strategy to decrease snail density in China. Many previous studies have made clear that vegetation (biotic factors) and soil (abiotic factors) were the basic requirements for snail survival on beaches. Moreover, a lot of research on snail control has been focused on the specific influencing environmental factors for snail survival, such as the vegetation community structure, species composition, diversity index, and the physical and chemical properties of the soil. Most of the existing research has studied the influence of a single factor on snail population density. Conversely, there have been only a few studies focused on the food sources and food composition of the snails. The current research situation on snail control has indicated that the mechanisms underlying ecological snail control have not been systematically characterized. The question of whether biotic or abiotic factors were more important in influencing snail survival remains unclear. Afforestation on beaches has significantly suppressed snail density in China so far. In this study, we proposed that the reduction of snail density was not affected by a single factor but by the interactions of multiple related factors introduced by afforestation. Moreover, different biotic and abiotic factors have significantly different effects on snail control. Therefore the goal of this study was to evaluate the relative importance and interactions of related biotic and abiotic factors on snail density. Methods: Four major vegetation communities: Sedge, Reed, Artificial poplar (3 years of age) and Artificial poplar (5 years of age), on the beaches of the Yangtze River in China were selected for vegetation and snail surveys, as well as for soil sampling. Structural Equation Model (SEM) analysis was used to assess the interactions of biotic and abiotic factors in the context of snail ecology. The soil properties were considered as abiotic factors, while algae of Chlorophyta, Cyanophyta and Bacillariophyta phyla were considered to be biotic factors. In the path analysis, the total effect between the variables was the sum of the direct and indirect effects.

**Results:**

The snail density had significant correlations with soil properties, such as water content, bulk density, capillary porosity and pH value, as well as with all three types of soil algae, *Chlorophyta*, *Cyanophyta*, and *Bacillariophyta*. Snail density had a direct negative relationship with capillary porosity and soil bulk density, an indirect negative relationship with soil pH value and an indirect positive relationship with soil water content via soil algae. Meanwhile, as an important food source for the snail, the *Chlorophyta*, *Cyanophyta* and *Bacillariophyta algae* had a significant positive correlation with snail density. High soil pH had a negative impact on *Chlorophyta*, *Bacillariophyta*, while soil water content had a positive impact on *Chlorophyta*, and soil bulk density had a negative impact on *Cyanophyta*. In addition, the soil pH value and soil bulk density both had negative correlations with soil water content.

**Conclusion:**

Afforestation of the beach environment can significantly reduce the snail population density by altering ecological factors. Soil algae (biological factors) might be the key element that drives ecological snail control. As important habitat determinants, the impact of the properties of the soil (non-biological factors) on the snail population was largely mediated through soil algae.

## Author summary

Schistosomiasis is the second largest tropical parasitic disease in the world after malaria. By the end of 2018, the areas in which schistosomiasis is endemic in China are distributed across 12 provinces (municipality and autonomous region). Among these areas, 5 provinces, including Shanghai, Zhejiang, Fujian, Guangdong and Guangxi, have continued to consolidate achievements in schistosomiasis elimination. Sichuan Province has achieved transmission interruption and 6 provinces of Hunan, Hubei, Anhui, Jiangxi, Yunnan, Jiangsu have achieved transmission control. *Oncomelania hupensis* (Snail) is the only intermediate host for the parasite schistosomiasis *japonicum*, so snail control is an important measure to control the spread of schistosomiasis. So far, the area the snail inhabits in China is 3.63 billion m^2^, with more than 95% of this distributed along the beaches of Yangtze River. Forestry schistosomiasis prevention projects has effectively reduced the snail habitat area and decreased snail population density through afforestation of these areas in China. However, the key factors that inhibit snail population growth by afforestation remain unclear. We propose to investigate the interactional relationship between biotic and abiotic factors introduced by afforestation on the distribution of the snail population. The results revealed that the number of soil algae is the important biological element determining snail distribution. Abiotic factors, such as soil properties indirectly impact on the snail population through a direct influence on soil algae. Of these, the pH value, water content, capillary porosity, bulk density interact with the algae content (*Chlorophyta*, *Cyanophyta* and *Bacillariophyta*) to decrease snail density through afforestation.

## Introduction

Schistosomiasis is one of the most important neglected tropical diseases (NTD), affecting an estimated 240 million people in 76 countries in Asia, Africa, and Central and South America [1–4]. *Schistosoma japonica*, prevalent in Indonesia, the Philippines and China, is one of five *Schistosoma* species that are of major public health concern and is the only endemic species in China [5,6]. The amphibious fresh-water snail, *Oncomelania hupensis*, is the sole intermediate host for *S*. *japonica* [7,8], where transition from larval form miracidium to larval form cercaria occurs during the life cycle of the parasite [9]. Therefore, snail control has been recognized as a strategy to impede schistosomiasis transmission [7,10,11]. In China, more than 90% of *Oncomelania* snails live in the beach areas of marshland and lake regions along the Yangtze River, as well as around the Poyang and Dongting Lakes [12].

Application of molluscicides, e.g. *niclosamide*, used to play an important role in controlling schistosomiasis in endemic areas [13]. However, these chemicals are toxic to aquatic animals and harmful to the environment [14], and are found to be less effective in low transmission endemic areas of schistosomiasis [10].

Several studies in different countries have shown that vegetation is necessary for snail survival. Vegetation provides shelter from excessive sunlight, support for egg hatching and food such as soil algae for the snail population [15–19], hence vegetation removal and cleaning is an effective measure for snail control and widely used in Morocco and Sudan in Africa. However this method is not sustainable as the reduction in snail density is short-lived [20], since the snail population can re-colonize the habitats following vegetation recovery [21]. Several studies have sought to achieve a sustainable snail control strategy via altering vegetation conditions [22], a concept of ecological snail control developed in the 1980s in China. During the 1980s-1990s, Peng [23] found that establishment of poplar plantations replacing the original vegetation, such as Sedge and Weed communities decreased snail density in beach areas. Snail control by afforestation (Forestry Ecological Projects for Snail control) has many advantages that not only provide a sustainable effect on schistosomiasis prevention without causing environmental pollution, but also serve powerful ecology functions and deliver great economic benefit.

In China, there have been three developmental phases of Forestry Ecological Projects for Snail control dating from the 1980s: scientific research; experiment and demonstration; as well as construction. By 2015, more than 5.18 billion hectares of forest, mainly poplar, have been planted for schistosomiasis prevention in the provinces of Hunan, Hubei, Jiangxi, Anhui, Jiangsu, Yunnan and Sichuan. Most of these forests are distributed along the beach areas of the Yangtze River [24]. Between 1986 and 2000, funds to support this research and demonstration work have come from Chinese state investment. In 2006, the State Forestry Administration of China implemented the “National Forestry Ecological Projects for Snail control planning (2006–2015)” which were devoted to snail control and schistosomiasis prevention. According to the most recent “Forestry Schistosomiasis Control Program (2016–2020)”, the investment for these projects has been China Yuan (CNY) 18000 per hectare. Over the past forty years, the projects have covered 194 counties in 7 provinces in the Yangtze River area of southern China [25].

Investigations into the mechanisms of snail control by afforestation have been conducted over the past several decades. Wu [26] reported that Sedge and Reed communities were beneficial for snail survival in marshland and lake regions along the Yangtze River. Planting artificial poplar forests were found to have negative impacts on the snail population by altering the soil environment [23], with the negative effect of the canopy environment increasing with plantation age [27,28]. Forest plantations can affect snail ecology by influencing soil humidity, pH, as well as the oxygen and algae contents [19,29–34]. However, most of these studies largely focused on a single factor affecting the snail population density, with the exception of a few recent studies exploring the impact of multiple interacting factors on snails [35,36]. Although the application of ecological snail control measures such as the “Forestry Ecological Projects for Snail control” has effectively reduced snail density, the mechanisms underlying this control have not been systematically characterized. Indeed, how biotic and abiotic factors interact to influence snail growth remains largely unexplored [37]. Improved knowledge will help to inform optimal snail control by afforestation.

Ecology focuses on the study of the relationships between biology and the environment. The key point of this study is a typical ecological issue that is to define the mechanism of snail control by afforestation, in other words, what are the interactions between snail density and the environmental changes introduced by afforestation. Since the essence of ecology is complex and consists of multiple interacting processes, statistical methods are particularly important for quantifying and defining relationships in ecological research. With the development of ecological methods and complex study environments, the univariate statistical method is no longer suitable to clearly define the complexity of the interactions that occur within ecosystems. As a multivariate statistical method, the Structural Equation Model, SEM can provide a new approach or direction to define complicated ecological relationships [38]. This study aims to use a Structural Equation Modeling (SEM) approach to evaluate multivariate hypotheses and identify the direct and indirect relationship pathways and causal associations between snail density and ecological factors, such as soil properties and algae content, following afforestation in the beach land along the Yangtze River in China. The results will offer important insights into the mechanisms underlying snail control through afforestation and lay the foundation for the development of improved snail control methods to eliminate schistosomiasis. S4 Fig shows the conceptual flow chart for this study describing the research ideas and frameworks.

## Methods

### Study sites

The Junshan District of Yueyang, in the Hunan Province (113°0’ E. 29°43’ N), located west of the Yangtze River and northeast of Dongting lake (S2 Fig), has a typical subtropical humid climate. The average annual temperature is 16.4–17°C and the annual amount of solar radiation is 418.68–460.55 kj/m^2^. In addition, the average annual sunlight duration is 1600–1850 h and accumulated temperature over 10°C is 5200–5350°C. The soil is mainly waterloggogenic paddy soil. The Junshan study sites are located in the outside-embankment areas of the Junshan district that are typical of schistosomiasis endemic areas [39].

### Data sources

The map of S2 Fig was downloaded from the Resource and Environment Science and Data Center(http://www.resdc.cn).

### Sample plots

The field investigation area was located in the outside-embankment beach of the Yangtze River in Junshan (S2 and S3 Figs) and the field sampling sites consisted of four major vegetation types: communities of Sedge, Reed, young artificial poplar plantation (3 years old), matured artificial poplar plantation (5 years old). Sedge and Reed are local vegetation communities suitable for snail survival [40], while artificial poplar plantations belong to the Forestry Ecological Projects for Snail control (Table 1).

**Table 1:** Characteristics of sample plots in the four vegetation communities.

Plot	Geographical coordinates	Elevation/m	Dominant species	Herbaceous vegetation community characteristics		
				Average height (cm)	Density (one/m^2^)	Coverage (%)
Sedge community	113°1‘1”E, 29°23’18" N	27	*Carex brevicuspis*	39.17±9.52	205.63±56.48	90.63±10.14
			*Myosoton aquaticum (L*.*) Moench*			
			*Astragalus sinicus L*			
Reed community	113°1‘12”E, 29°22’48" N	28	*Phragmites communis*	53.12±10.08	105.57±47.22	94.86±0.35
			*Mazus japonicus*			
			*Astragalus sinicus* L			
Herbaceous community under artificial poplar (3 years old)	112°58‘48”E, 29°28’48" N	29	Alternanthera *philoxeroides*	40.86±29.02	105.22±32.46	72.22±11.33
			*Apium leptophyllum*			
			*Purslane Speedwell*			
Herbaceous community under artificial poplar (5 years old)	112°54‘36”E, 29°31’12" N	29	*Leonurus japonicus*	45.67±13.81	77.44±42.12	65.11±20.40
			*Roegneria kamoji Ohwi*			
			Cnidium monnieri (Linn.) Cuss			

First of all, the area of every investigated vegetation type was 1 ha (100m ×100m). Each vegetation plot type had 3 replicate sample plots, consisting of 30 small plots separated by 10m (S1 Fig). In practice, the distance between every replicate sample plot was 20~30m. The principle of designating this distance was mainly to maintain as consistent a site condition as possible. The sample size was sufficient to represent the populations. Specifically, in the field investigation, each plot was designed to contain sample sizes to satisfy all three experimental components of snail survey, vegetation survey and soil sampling. After comprehensive consideration, the current sample size was found to be reasonable for this study. Under the experimental conditions, the current sample size meets the requirements for the Structural equation model [41,42].

## Sampling and investigation

### Vegetation survey

Investigation of the vegetation community composition was conducted in all the four types of environments recording the species name, average height, abundance, total coverage, and biomass. In every sample plot, there are 30 small plots (1m×1m) for the vegetation survey.

### Snail survey and sampling

Each small plot in the study was surveyed for snail population density using systematic sampling. All snails found in the plots were collected in separate bags [19]. In the laboratory, 20 snails from each sample plot were dissected and the tissue preserved in centrifuge tubes containing 75% alcohol.

### Soil sampling

As described above, each small plot was subjected to sampling of the top layer of soil to a depth of 2 cm. A total of 100g for each soil sample was gathered and taken back to the laboratory for further study.

### Soil algae sampling

Samples of soil algae were also collected in a similar manner to the soil sampling described above. Samples were stored in centrifuge tubes with distilled water at 20–30°C. The quantity per sample of soil algae was 50g.

### Measurements

The physicochemical characteristics and algae content of the soil were measured following the methods of Fang, Jin and Jiang, as shown in Table 2 [43–45].

**Table 2:** Methods for measurement of soil physiochemical characteristics and algae.

Index of Properties		Methods
Soil physical	Soil water content	Cutting-ring method
	Soil bulk density	
	Capillary porosity	
	Saturated soil water	
Soil chemical	Soil pH value	Potentiometry
	Organic matter	Oxidation of potassium dichromate-external heating
	Total nitrogen	Semi-micro kjeldahl method
	Total phosphorus	Colormetry
	Total potassium	Flame photometry
	Hydrolysable nitrogen	Alkaline hydrolysis diffusion
	Available phosphorus	Hydrochloric acid and sulfuric acid extraction
	Available potassium	Flame photometry
Soil algae	Cyanophyta	Cultured in BG11 medium and counted under microscope
	Bacillariophyta	Cultured in CSI medium and counted under microscope
	Chlorophyta	Cultured in BG11 medium and counted under microscope

### Data analysis

The relationship between snail density and the soil physicochemical characteristics and algae content was surveyed using linear correlation analysis with SPSS19.0. In the linear correlation analysis, all tests were two-sided and the level of statistical significance was set at 0.05.

The structural equation model (SEM), also called the latent variable model [46], was developed to analyze the interactions of biotic and abiotic factors in the context of snail ecology. The soil physicochemical properties were considered as abiotic factors, while the biotic factor was the soil algae content, where *Chlorophyta*, *Cyanophyta* and *Bacillariophyta* were the three main algal phyla found in soil samples. In short, SEM is a multivariate statistical method that integrates both factor and path analyses, which can simultaneously define the correlation between multiple factors [47]. In addition, the function of the structural equation model was designed to include evaluating the relationship strength of each factor within the context of fitting the data to the whole model [38].

AMOS 24 was used for path analysis between snail density and related biotic and abiotic factors. Soil properties were set as the external variables, while snail density was set as the internal variable and the soil algae content of *Chlorophyta*, *Cyanophyta* and *Bacillariophyta* were the intervening variables, set as both external and internal variables. Based on theoretical knowledge and an *a priori* test, we assumed a conceptual model in which the soil properties affect soil algae content, which in turn, affects the snail density (S5 Fig). A maximum likelihood estimation method was used to compare the SEM models with the experimental observations. Goodness of fit for the model to the data was determined using the criteria where χ^2^ was not significant (p>0.05), RMSEA (root mean squared error of approximation) < 0.08 and TLI (Tucker-Lewis Index) > 0.95 [48]. In order to satisfy the requirement that SEM is applied to a normal distribution, all data were standardized using the Z-score [49](S4 Table). The model was iteratively fitted multiple times to the data in order to finally reach all the adaptable standards, with multiple corrections to release paths without significant effects. The goodness of fit values for the correction model is shown in S1 Table.

In this study, AMOS 24 was used as the tool to build the Structural Equation Model (SEM) describing the interaction between snail density and the related factors. Based on theoretical knowledge and standards of model fit, the path relationships of SEM have been adjusted multiple times to fit all the adaptable standards (S1 Table).

In all fitting parameters, Chi-square degrees of freedom (χ2/df) is one of the most important indexes to judge the fitness of SEM which evaluates the deviation between the actual observed sample data and the model described value. Similarly, the significant probability value (P) represents the degree to which the covariance matrix of samples matches the matrix implicit in the theoretical model. The value of P>0.05 indicates that the sample data satisfactorily matched the theoretical model. In this study, fitting parameters for χ2/df = 0.935<3 and P = 0.515>0.05 clearly show that the variable data for soil properties and algal content, as well as for snail density are well fit with SEM. This means that use of SEM to analyze experimental data can provide a good explanation about the interaction between snail density and related factors.

## Results

### Variation of snail density with vegetation type

In order to determine the effect of vegetation type on snail density, a snail survey was carried out in four different vegetation types: Sedge community; Reed community; Artificial poplar community (3 years old) and Artificial poplar community (5 years old). S3 Table shows the number of snails found in each small plot within the larger plots for each vegetation type.

**Fig 1:**
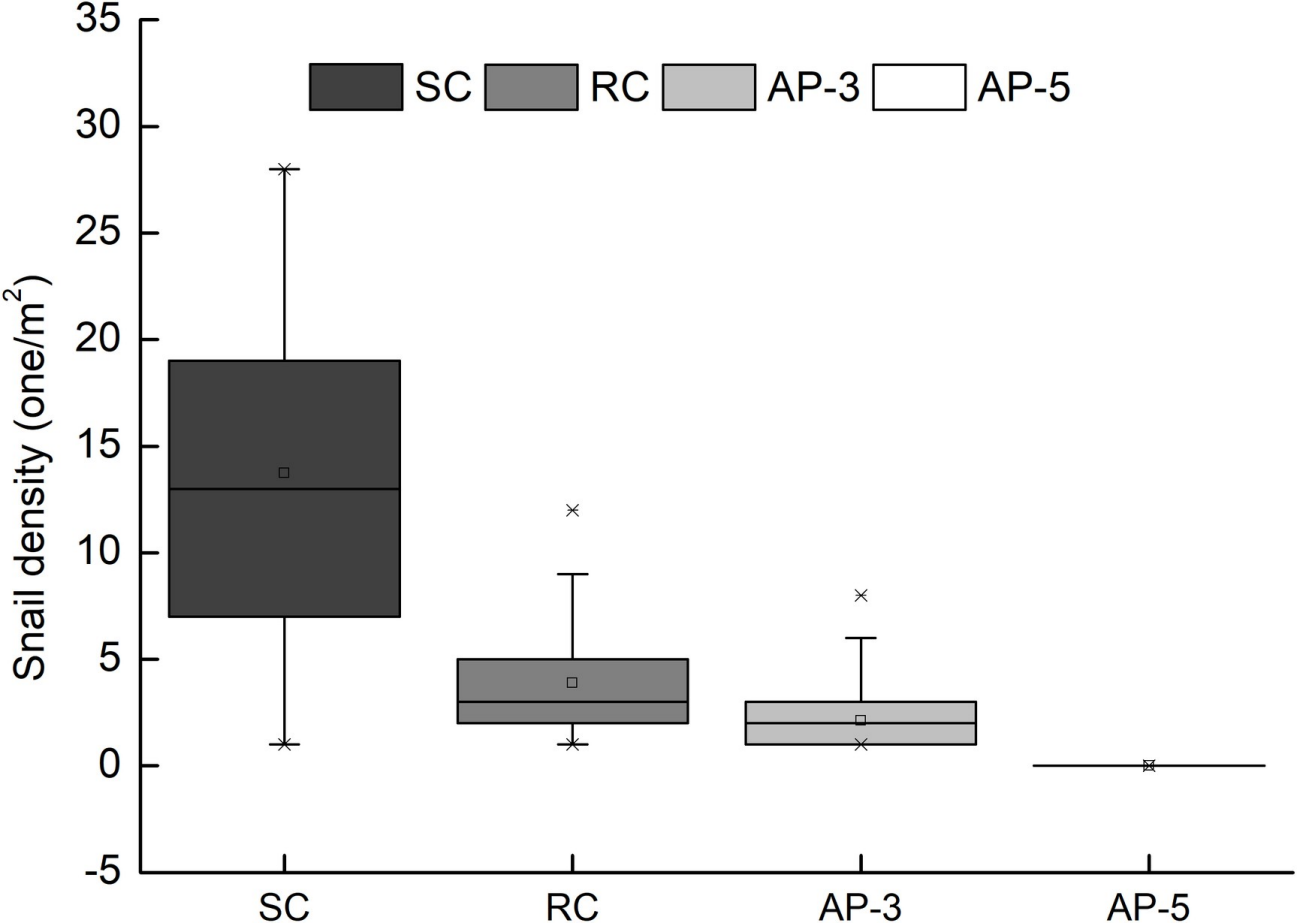
Results of snail density in the four typical vegetation types (n = 30 for each vegetation type). SC: Sedge community; RC: Reed community; AP-3: Artificial poplar community (3 years old); AP-5: Artificial poplar community (5 years old).

Fig 1. shows that among these four typical vegetation communities, the order of the average snail density from high to low is as follows: Sedge community (12.74±7.57 one/m^2^) > Weed community (3.89±2.75 one/m^2^) > Artificial poplar community (3 years old) (2.12±1.25 one/m^2^) > Matured artificial poplar community (5 years old) (0 one/m^2^). In general, the average snail density under artificial poplar forests was lower than those found in native vegetation communities of Sedge and Reed. Of particular note, no snails were found in the matured artificial poplar forest older than 5 years (Fig 1).

### Correlation between snail density and soil properties, soil algae

Linear correlation analysis showed that each type of soil algae was significantly correlated with snail density, including *Chlorophyta* (r = 0.917, p<0.01), *Cyanophyta* (r = 0.860, p<0.01) and *Bacillariophyta* (r = 0.877, p<0.01) (S2 Table). Among soil physicochemical properties, only four properties showed significant correlation with snail density (p<0.05). Specifically, snail density had a positive correlation with soil water content (r = 0.661, p<0.01), but a negative correlation with soil bulk density (r = -0.543, p<0.05), capillary porosity (r = -0.442, p<0.05) and soil pH value (r = -0.405, p<0.05). Other indices of soil properties did not show any significant correlation with snail density. Meanwhile soil water content showed a significant positive association with the presence of soil algae *Chlorophyta* (r = 0.560, p<0.05), *Cyanophyta* (r = 0.659, p<0.05), *Bacillariophyta* (r = 0.450, p<0.05). Soil bulk density had a significant negative correlation with the presence of *Cyanophyta* (r = -0.687, p<0.01). Capillary porosity was negatively related to all three algae phyla of *Chlorophyta* (r = -0.416, p<0.05), *Cyanophyta* (r = -0.352, p<0.05), *Bacillariophyta* (r = -0.435, p<0.05), while soil pH value also had a negative relationship with the presence of *Chlorophyta* (r = -0.451, p<0.05) and *Bacillariophyta* (r = -0.474, p<0.05). The concentration of free potassium had a negative relationship with the presence of *Bacillariophyta* (r = -0.446, p<0.05) and *Cyanophyta* (r = -0.529, p<0.05). Other indices for soil properties did not show significant correlations with snail density or soil algae content. The correlation analysis showed that soil algae had a closer relationship with snail density while the soil properties were more closely associated with soil algae content (S2 Table).

### Structural equation model (SEM)

Based on the correlation analysis (S2 Table), seven factors were shown to have significant correlation with snail density. Additionally the soil properties and soil algae content had significant correlations with each other. Consequently, these seven factors were used to construct the structural equation model that relates snail density with the related biotic and abiotic factors.

According to the existing research results and correlation analysis, in this study, snail density was found to be significantly influenced by the soil properties of water content, capillary porosity, bulk density, pH value and content of algal phyla *Chlorophyta*, *Cyanophyta* and *Bacillariophyta*. The basic statistics are shown in Table 3.

**Table 3:** Basic statistics of Snail density, Soil properties and Soil algae.

Indices			Minimum	Maximum	Mean±SE
Snail density	SC		1.00	28.00	12.74±7.57
	RC		1.00	12.00	3.89±2.75
	AP-3		1.00	8.00	2.12±0.59
Soil properties	Soil water content (%)	SC	32.19	44.67	38.68±0.61
		RC	22.42	29.03	24.84±0.35
		AP-3	25.04	29.51	27.10±0.22
		AP-5	28.99	32.38	30.66±0.80
	Capillary porosity	SC	0.46	0.52	0.50±0.02
		RC	0.39	0.45	0.42±0.19
		AP-3	0.40	0.43	0.42±0.06
		AP-5	0.37	0.51	0.44±0.03
	Soil bulk density (g/cm^3^)	SC	1.11	1.39	1.25±0.14
		RC	1.41	1.59	1.52±0.10
		AP-3	1.20	1.56	1.34±0.15
		AP-5	1.21	1.49	1.34±0.07
	Soil pH value	SC	6.14	6.91	6.31±0.26
		RC	7.01	7.10	7.05±0.05
		AP-3	7.02	7.65	7.33±0.33
		AP-5	7.49	7.64	7.60±0.05
Soil algae	*Chlorophyta* (one/mg soil)	SC	232.00	1100.00	506.83±134.88
		RC	306.00	547.00	433.67±69.94
		AP-3	92.00	308.00	243.50±20.66
		AP-5	91.00	103.00	96.00±28.77
	*Cyanophyta (one/mg soil)*	SC	0.00	138.00	52.67±21.94
		RC	5.00	33.00	17.67±8.19
		AP-3	6.00	50.00	22.00±14.05
		AP-5	24.00	35.00	30.26±23.74
	Bacillariophyta (one/mg soil)	SC	0.00	150.00	50.83±21.64
		RC	39.00	71.00	52.33±9.62
		AP-3	25.00	45.00	33.33±6.01
		AP-5	0.00	15.00	7.00±7.55

**SE**: the abbreviation for standard error.

**Fig 2:**
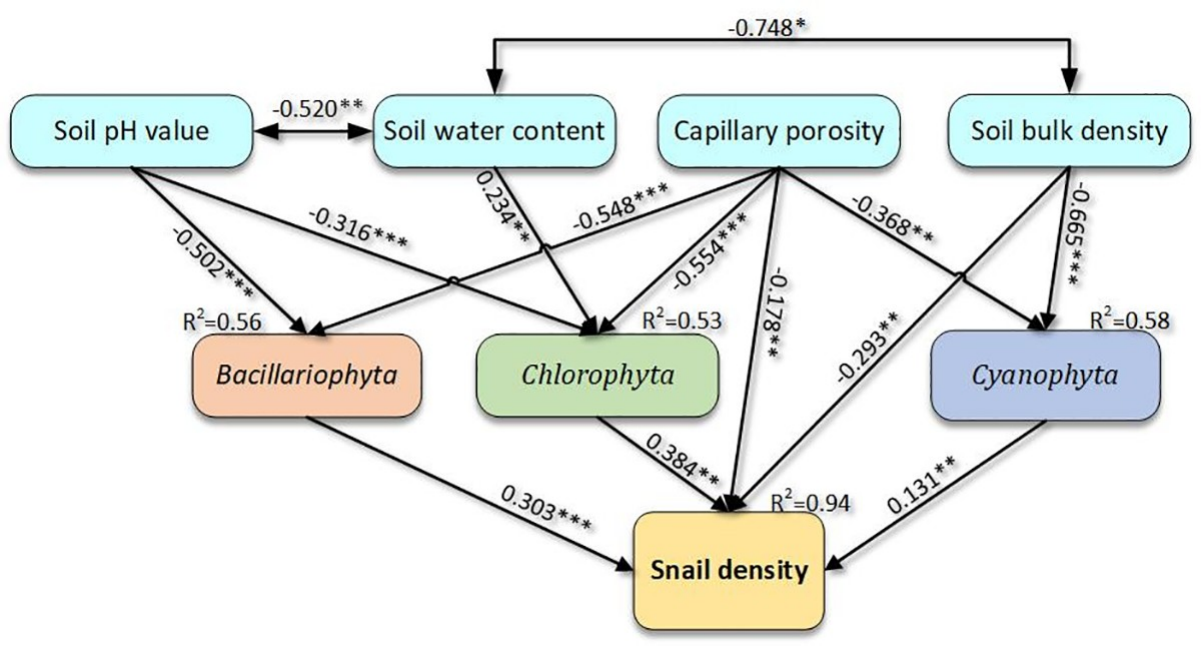
SEM of snail density and related factors.

Capillary porosity was shown to have a negative effect on snail density (path coefficient = -0.178, P<0.01) (Fig 2), likely mediated through a negative impact on the soil algae of *Chlorophyta*, *Cyanophyta* and *Bacillariophyta*, found to have respective path coefficients of -0.554 (P<0.001), -0.368 (P<0.01) and -0.548 (P<0.001). The indirect impact on snail density from capillary porosity was found to have a path coefficient of -0.427 through the three types of soil algae. The total effect of capillary porosity was found to be -0.605 (Table 4). Similarly, soil bulk density had negative effects on both snail density and *Cyanophyta*, with coefficients of -0.293 (P<0.01) and -0.665 (P<0.001), respectively. The coefficient for the indirect effect on snail density was -0.087 while the coefficient for the total effect coefficient was -0.381. Meanwhile the soil pH value had negative effects on the soil algae *Chlorophyta* and *Bacillariophyta* (path coefficient = -0.316 (P<0.001) and -0.502 (P<0.001)) but only an indirect effect on snail density with a coefficient of -0.274 (Table 4). Soil water content only had a direct positive effect on *Chlorophyta* (path coefficient = 0.234 (P<0.01)) and a weak positive indirect influence on snail density with a coefficient of 0.090. In addition, the three soil algae phyla, *Chlorophyta*, *Cyanophyta* and *Bacillariophyta* all had positive effects on snail density with path coefficients of 0.384 (P<0.01), 0.131 (P<0. 01) and 0.303 (P<0.001), respectively. In the SEM analysis, the total of seven factors jointly explained 94% (R^2^ = 0.94) of the variance for the disparities found in snail density between the different vegetation types (Fig 2). As the intervening variables, soil algae were influenced by other soil properties and thus also had an effect on snail density. Therefore, the soil properties of soil water content, soil pH value, soil bulk density and capillary porosity all have various indirect effects on snail density. In this study, soil water content had negative correlations with both soil pH value and soil bulk density with coefficients of -0.520 (P<0.01) and -0.748 (P<0.05), respectively.

**Table 4:** Standard impact coefficients of SEM for Snail density and related factors.

Variables	Standardized Direct Effects				Standardized Indirect Effects				Standardized Total Effects			
	X13	X14	X15	X1	X13	X14	X15	X1	X13	X14	X15	X1
X2			0.234					0.090			0.234	0.090
X5		-0.502	-0.316					-0.274		-0.502	-0.316	-0.274
X3	-0.665			-0.293				-0.087	-0.665			-0.381
X4	-0.368	-0.554	-0.548	-0.178				-0.427	-0.368	-0.554	-0.548	-0.605
X13				0.131								0.131
X14				0.303								0.303
X15				0.384								0.384

X1: Snail density; X2: Soil water content; X3: Soil bulk density; X4: Capillary porosity; X5: Soil pH value; X13: *Cyanophyta;* X14: *Bacillariophyta;* X15: *Chlorophyta*.

## Discussion

A healthy snail population requires a sheltered space and optimal microenvironment conditions provided by the vegetation communities in which they exist [18]. Previous surveys indicated that there is negligible snail survival on an unvegetated beach along the Yangtze River, even if other environmental conditions are appropriate [19]. Snail density was found to be very low when vegetation coverage was less than 20%, but increased linearly as vegetation coverage increased from 20%-60% [31]. Wu [25] and Jiang [50] also reported that the optimal vegetation coverage for snail growth was 35%-100%, with an average height 15-40cm. In this study, although vegetation height and coverage in 3-year old plantations were found to be suitable for low-density snail survival, no snails were found when the artificial poplar forest was more than 3 years old. This cannot be reasonably explained by previous research results. Here, our results implied that it was unlikely that the characteristics of the vegetation community had direct influence on snail density. Different vegetation communities are known to modify soil properties and algae communities, thus potentially have an indirect effect on snail density [18]. This study emphasized the direct effect of four soil properties on snail density, namely, water content, capillary porosity, pH value, and bulk density.

Soil is also an essential condition for snail survival. Indeed, snail populations were shown to be sensitive to changes in topsoil properties [19]. For example, soil water content is a limiting factor for snail survival [51]. In this study, soil water content had a direct positive effect on *Chlorophyta*, the main component of soil algae, but had no direct impact on snail density (Fig 2 and Table 4). The water content in soil samples was found to vary from 22% to 44% in this study, which, based on previous data, was thought to be sufficient for snail survival. Surprisingly, this was not found to be the major limiting factor on snail survival in all vegetation communities. In this study, the presence of *Chlorophyta* in topsoil became the main factor affected by soil water content, since abundant soil water supports soil algae growth [30].

The effect of soil capillary porosity on snail density may result from soil oxygen content, since high capillary porosity decreases the oxygen content in soil [34]. In this study, the oxygen content in topsoil was not optimal for snail growth and thus negatively influenced snail density. High capillary porosity can also affect soil texture and thus limit the distribution of soil algae (Table 4).

Snail populations prefer slightly acidic conditions with pH values of 5.2–6.5 and soil alkalization is not beneficial to snail survival [52]. Planting artificial poplar communities resulted in an increased soil pH value, however, the influence on snail density was not significant for pH values 6.14–7.65. Indeed, algae are more sensitive to soil pH value changes, with high pH values usually being damaging to algal survival [33,53]. In this study, the soil pH value was found to negatively affect both *Chlorophyta* and *Bacillariophyta* (Fig 2), and thus indirectly affect snail density.

A negative correlation was shown between snail population density and soil bulk density, indicating that the snail population prefers fluffy soil conditions [31]. It is difficult for the snail to live (e.g., burrow holes) in soil with high soil bulk density usually found during the winter season [19], as this type of soil has poor permeability and a sticky texture. The unicellular algae *Cyanophyta* prefers sandy soil over clay soil [30,54]. The results of the model also indicated a negative correlation between soil bulk density and *Cyanophyta* (Fig 2).

The four soil properties described above can also interact with each other to influence snail growth. The increase in soil bulk density can result in a decrease of the soil water content [55]. After periodic flooding, the process of soil drying will increase salinization and lead to an increase in the soil pH value [56]. In this study, soil water content had a significant negative correlation with bulk density and pH value, which were the main interactions in the Model (Fig 2).

As the most important primary producer on earth, algae produce energy through photosynthesis and supply a total amount of organic carbon that is seven times greater than higher plants. Dissection of snail digestive tracts determined that there were multiple algae in the intestines and stomach, including *Chlorophyta*, *Cyanophyta* and *Bacillariophyta* [19,29]. Microscopic examination has also found that there are various algae in soil with a high snail density [32]. Planting artificial poplar forests were found to remarkably decrease the number of soil algae including *Chlorophyta*, *Cyanophyta* and *Bacillariophyta*. The correlation model revealed that all three types of soil algae had a significant positive impact on snail density with a high path coefficient (Fig 2). The results indicated that variation of soil algae was more closely and directly related to the snail population than other soil properties. Soil algae, as the biological factor, might be the main limiting factor for snail survival under altered vegetation conditions.

Of particular note is the influence of environmental gradients moving from upstream to downstream. The study plots of four vegetation types have been all along the Yangze River beaches with similar elevation and topography. As S2 Fig shows, the spacing among 4 vegetation sample plots was near 4-5km. There is no doubt that environmental gradients extending from upstream to downstream occur within 15km of the sample plots and might be one of the influencing factors possibly limiting the value of this study.

Environmental changes, such as planting artificial forests, can affect multiple ecological processes and have complex interactions that influence snail population growth [57,58]. Exploring the direct and indirect relationships among these variables is important in ecological and environmental research. Here, a multivariate analysis method, Structural Equation Model (SEM), was used to reveal the relative effects of biological factors (soil algae) and non-biological factors (soil properties) on snail density following afforestation (Fig 2 and Table 4). We also determined the direct and indirect effect of soil properties and algae content on snail population density. This has provided new insight into understanding how environmental changes affect snail density. This information will facilitate the development of ecological snail control methods such as those used by the Forestry Ecological Projects for snail control and schistosomiasis elimination.

Previous studies have shown that optimal survival conditions for snails include a ground temperature of 20–25°C and 3600–3800 lux of sunlight intensity [59]. Herbaceous vegetation communities maintain a stable ground temperature and decrease sunlight intensity [21]. Afforestation on beach land resulted in the transition from a grassland ecosystem to a forest ecosystem [27]. Therefore, it is reasonable to speculate that afforestation possibly provides a strong effect of shade to lower ground temperature and reduced sunlight, which would have a negative effect on soil algae.

This study was conducted in the beach land along the Yangtze River and around the Dongting lake in the Junshan District of Yueyang, in Hunan Province. This area of lake and marshland regions accounts for 94.73% of the *Oncomelania* snail distribution in China, with two other environments, plains with a water-network and mountains, accounting for 0.06% and 5.21% respectively [60]. The limitation of this study is that the results presented here only represent lake and marshland regions and thus the mechanism of snail control by afforestation in other areas is still not clear.

Forestry Ecological Projects for snail control, as the main measure of ecological snail control in China, has strict operation and technical regulations. The key technical indexes include forestland selection, tree species selection, afforestation operation design and management of the forest stand [59]. Due to seasonal flooding, there are less plant species (including poplar, willow, mulberry and pecan) resistant to flooding and that can be widely used for afforestation in beach areas [61].

As an important measure for snail control in China, it is necessary to objectively evaluate afforestation. Forestry projects are an environment-friendly snail control measure that do not cause pollution and have long-term effects over many years. However, research in the field of wetland conservation believed that afforestation in beaches has partly influenced the integrity of native vegetation and the stability of wetland ecosystems by introducing foreign species. In fact, afforestation has a relatively reduced scope of application because of the strict operation and technical regulations [62]. Moreover, certain afforestation projects conflict with policies for wetland conservation. Therefore, forestry projects are restricted to being outside the wetland reserves aimed at protecting wetland ecosystems [25,63,64]. This restriction does not reduce the effectiveness of snail control.

Doubt about the source of the original snails after afforestation has always raised concerns. Unless encouraged by natural or human forces, snails prefer to stay within the boundaries of its breeding grounds rather than migrate long distances. The movement ability of snail is extremely weak. Previous studies indicated snails can move on wet soil surface nearly 2.7 m over 24 hours and as far as 19.5 m in 60 h, with almost no movement on dry soil surfaces [19]. Flooding is one of the main causes of snail spread. The investigations have indicated that the devastating 1998 floods in China increased the snail area along the river beaches of Nanjing by 2 million square meters, as well as increasing the snail area in Hubei province 7.33 fold.

In fact, the forested area has been much larger than the active radius of snail habitat. After afforestation, the snails were unlikely to move by themselves to nearby non-forested areas. Afforestation is an adverse environment that can effectively lead to the death of the original snails and no new snail survival. The snail survey data have shown that there were no snails in the 5-year old artificial poplar community (Fig 1). After years of prevention, schistosomiasis has become a low-transmission epidemic situation in China. However, there was no statistical data to compare schistosomiasis infection with snail density at the regional level. Nationwide statistics have shown that afforestation in epidemic areas decreased snail density by an average of 89.8%, while the schistosomiasis infection rate dropped by 51.0% up until 2016 [65].

## Conclusion

Here, our study showed that afforestation in beach land is able to effectively decrease snail density. Such methods are so effective that no snails were found in the matured artificial poplar forest that was 5 years old. The Structural Equation Model showed that snail density was directly decreased by soil capillary porosity and bulk density, with an indirect negative effect contributed by soil pH value and an indirect positive effect from soil water content. All soil algae, *Chlorophyta*, *Cyanophyta* and *Bacillariophyta*, directly affected snail density in a positive manner. High soil pH values were adverse for *Chlorophyta* and *Bacillariophyta*. Soil water content positively influenced *Chlorophyta* and soil bulk density negatively affected *Cyanophyta*. Our study emphasized that soil algae, as the important food source for *Oncomelania* snail hosts of *S*. *japonica*, might be the key element underlying ecological snail control by afforestation. Conversely, soil properties (non- biological factors) have a direct influence on soil algae and thus only indirectly influence snail population.

## Supporting information

S1 Table Fitting parameters of SEM. (TIF)

S2 Table Correlation analysis between snail density, soil properties and soil algae. (TIF)

S3 Table Statistical results of snail survey of different vegetation types (one/m2). (TIF)

S4 Table Normality Test. (TIF)

S1 Fig Layout of sample plots. (TIF)

S2 Fig The location of research areas in the Junshan district of Hunan. (TIF)

S3 Fig The photos of the four investigated vegetation types. (TIF)

S4 Fig A conceptual flow chart of snail control by afforestation. (TIF)

S5 Fig Conceptual model of SEM of snail density and related factors. (TIF)
