# Supplementary figures and images for: Afforestation suppresses Oncomelania hupensis snail density through influencing algae in beaches of the Dongting Lake
Source: PLoS Negl Trop Dis. 2021 Feb 4;15(2):e0009100. doi: 10.1371/journal.pntd.0009100 (PMC7888596; doi:10.1371/journal.pntd.0009100)

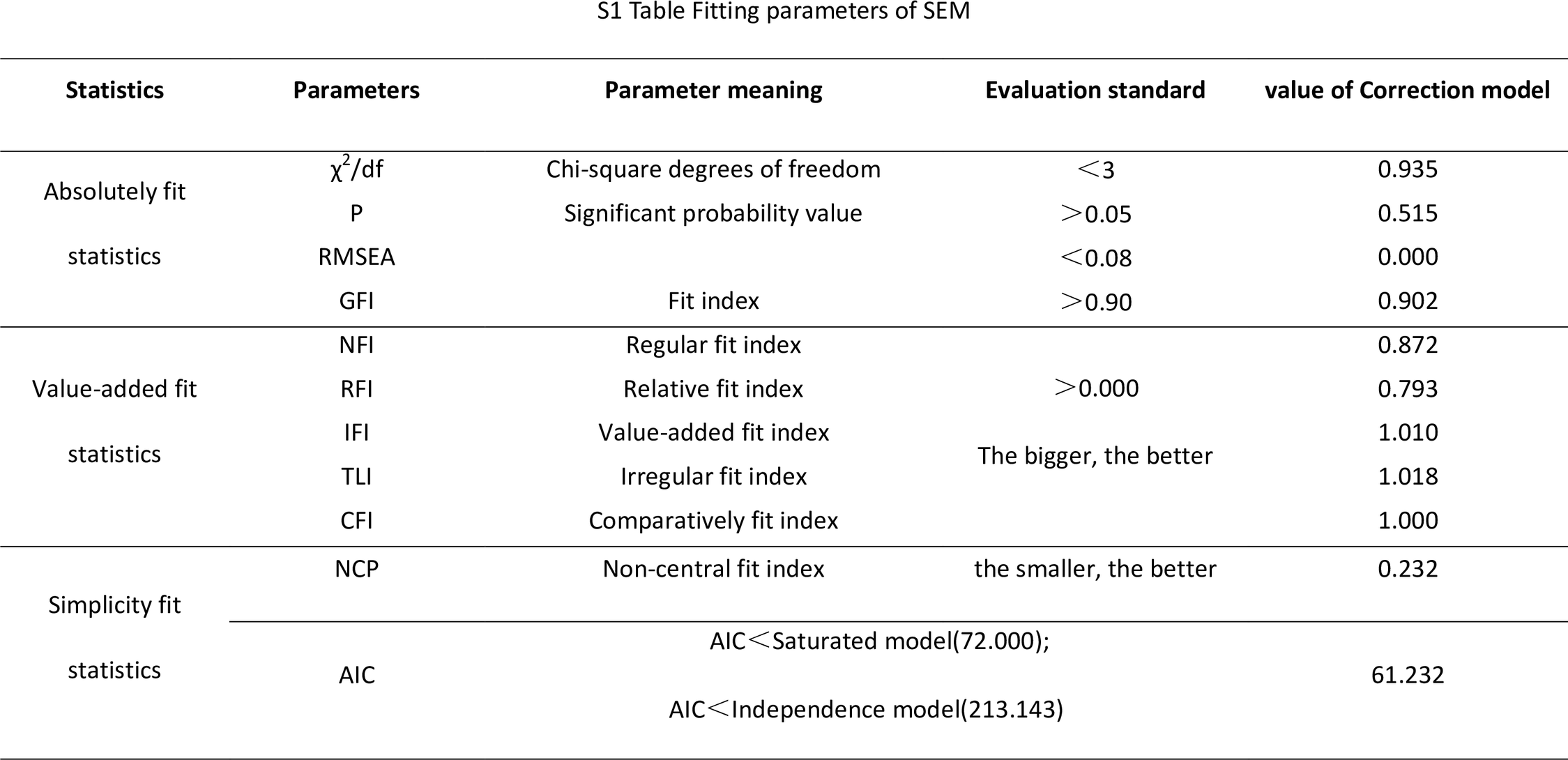

Supplement: S1 Table — (TIF) [file pntd.0009100.s001.tif]

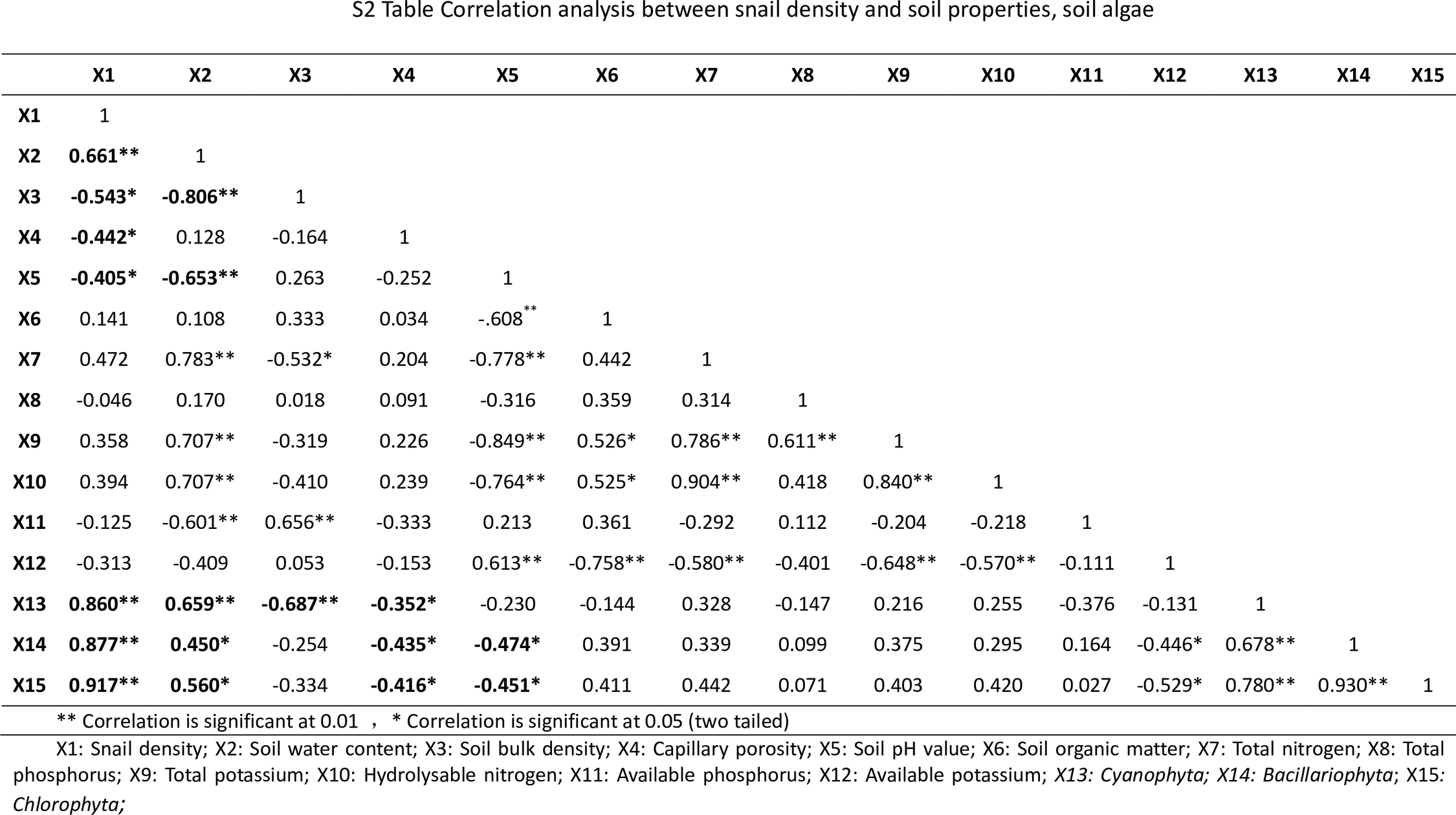

Supplement: S2 Table — (TIF) [file pntd.0009100.s002.tif]

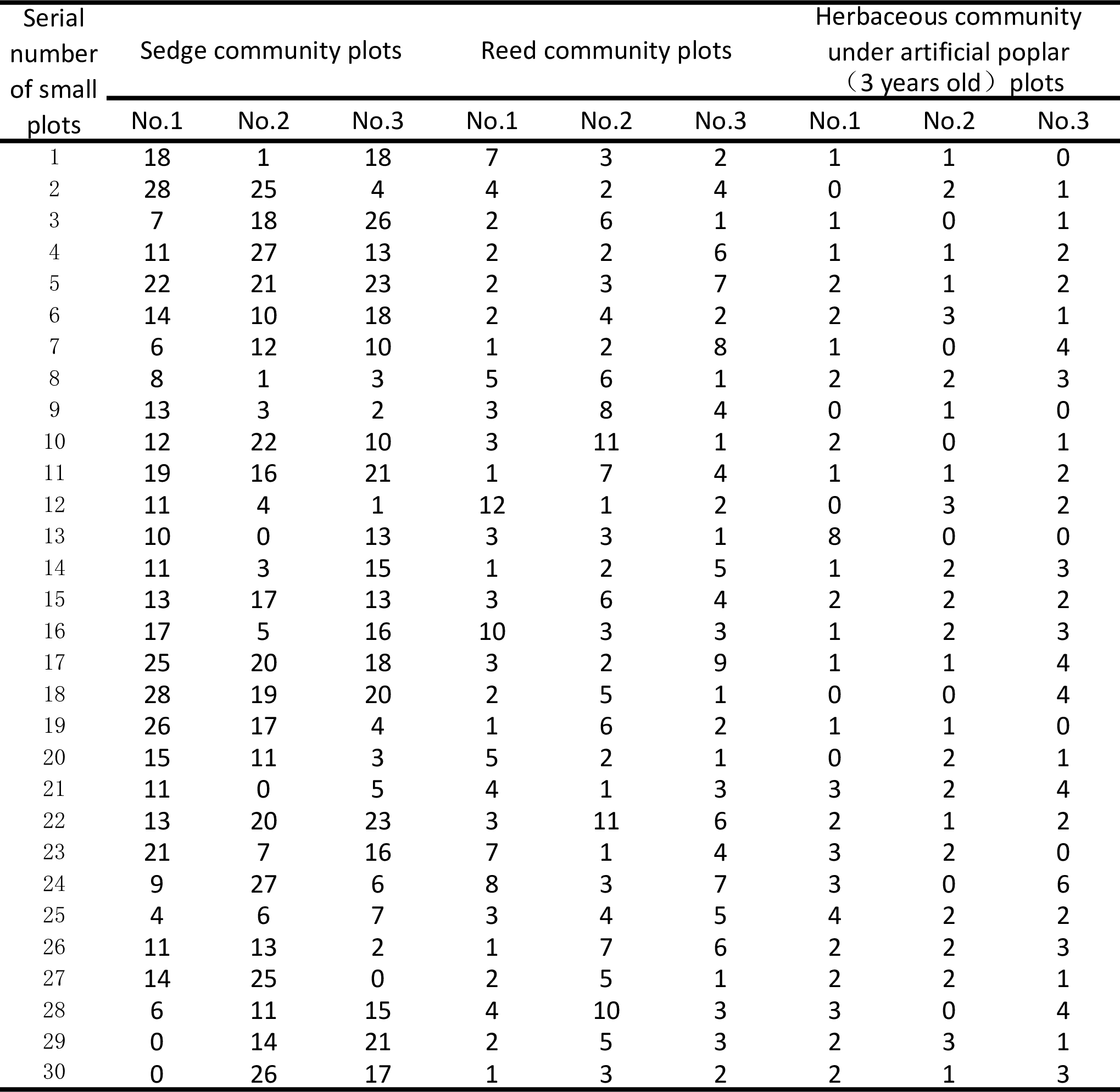

Supplement: S3 Table — (TIF) [file pntd.0009100.s003.tif]

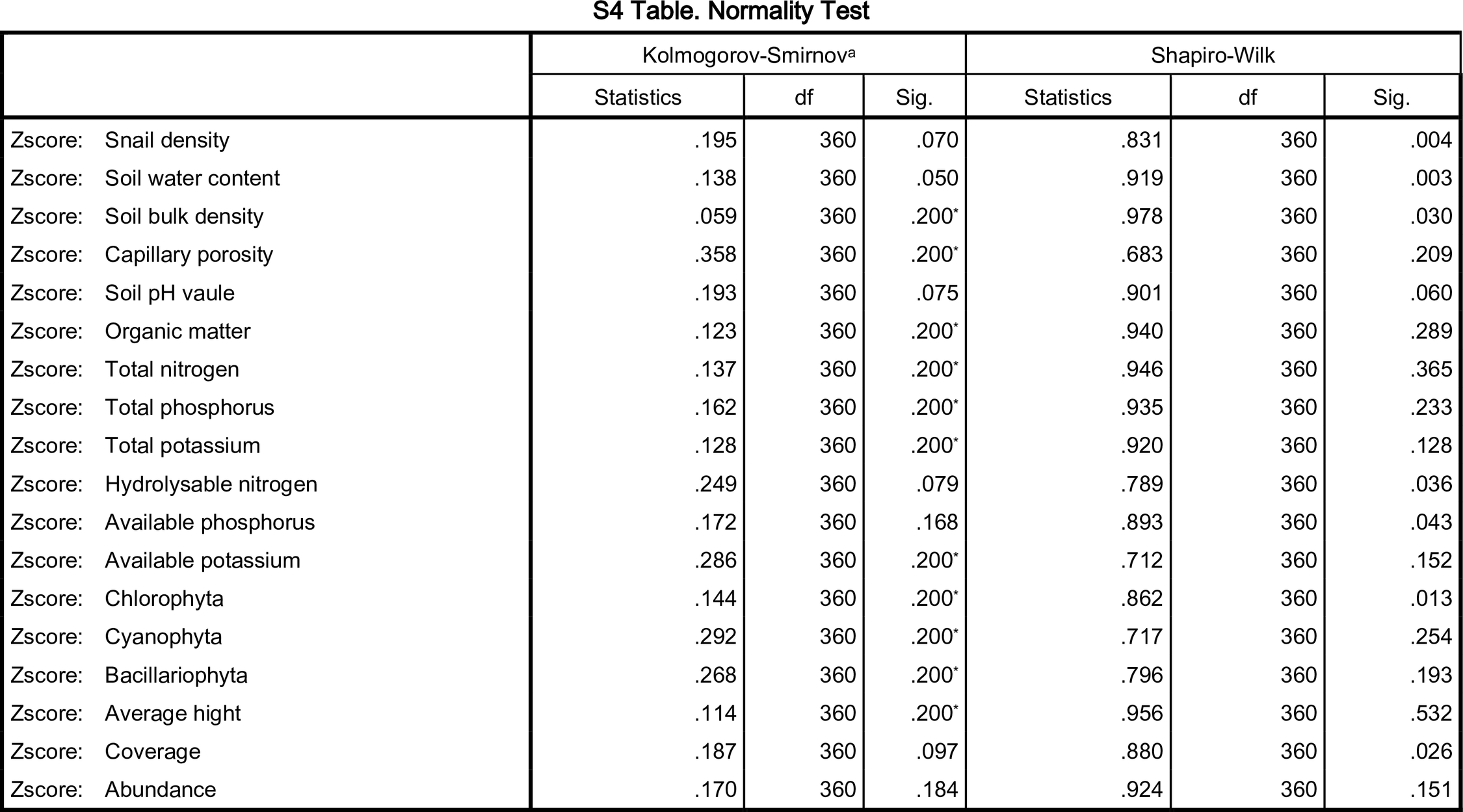

Supplement: S4 Table — (TIF) [file pntd.0009100.s004.tif]

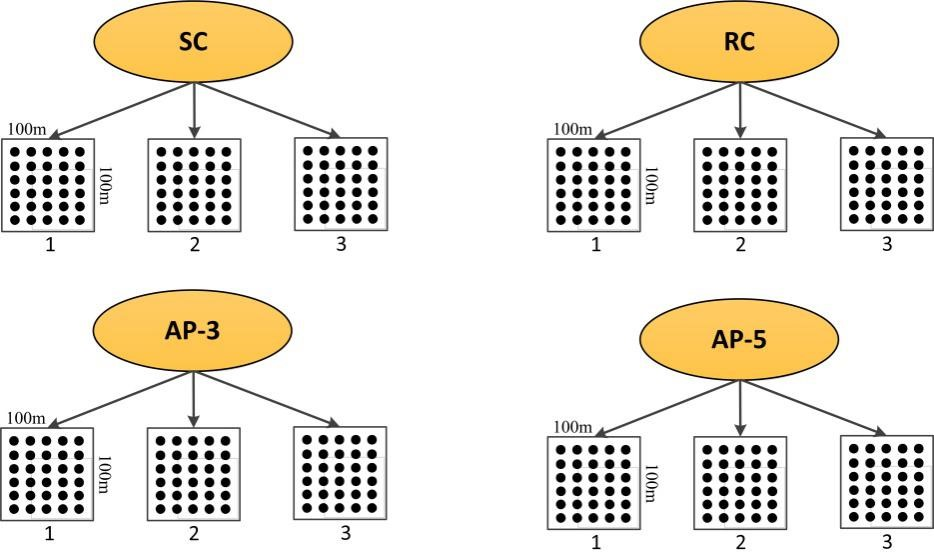

Supplement: S1 Fig — (TIF) [file pntd.0009100.s005.tif]

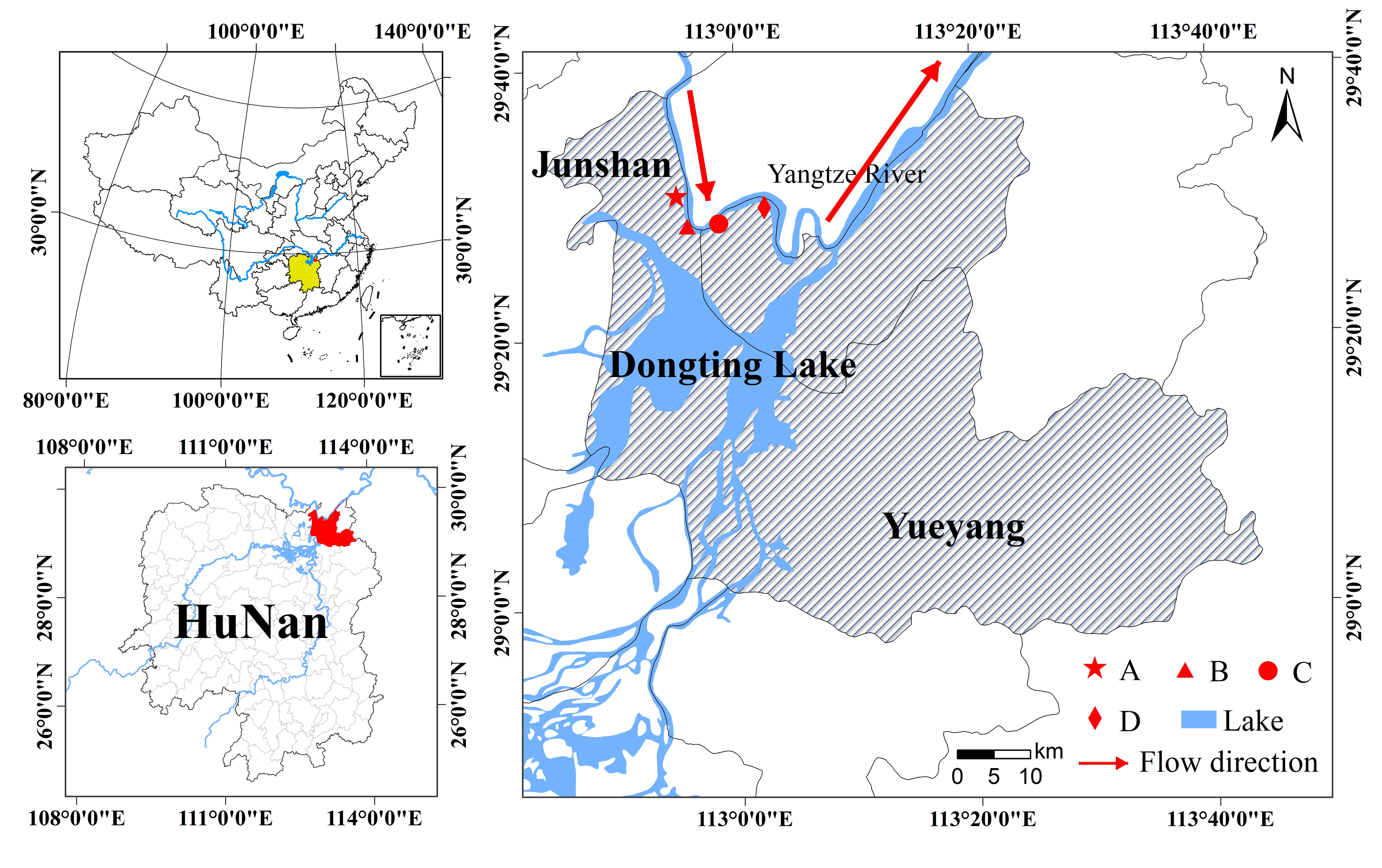

Supplement: S2 Fig — (TIF) [file pntd.0009100.s006.tif]

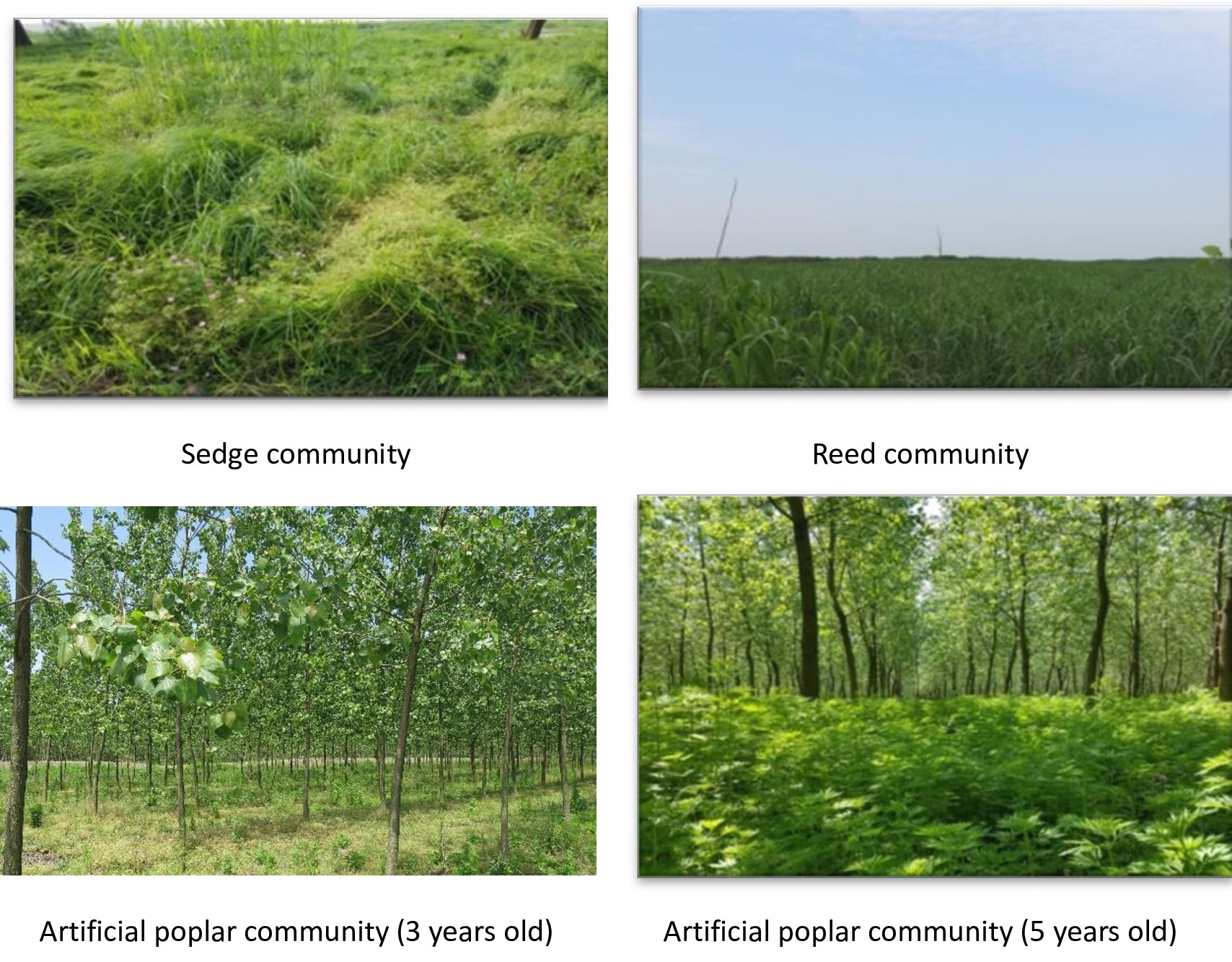

Supplement: S3 Fig — (TIF) [file pntd.0009100.s007.tif]

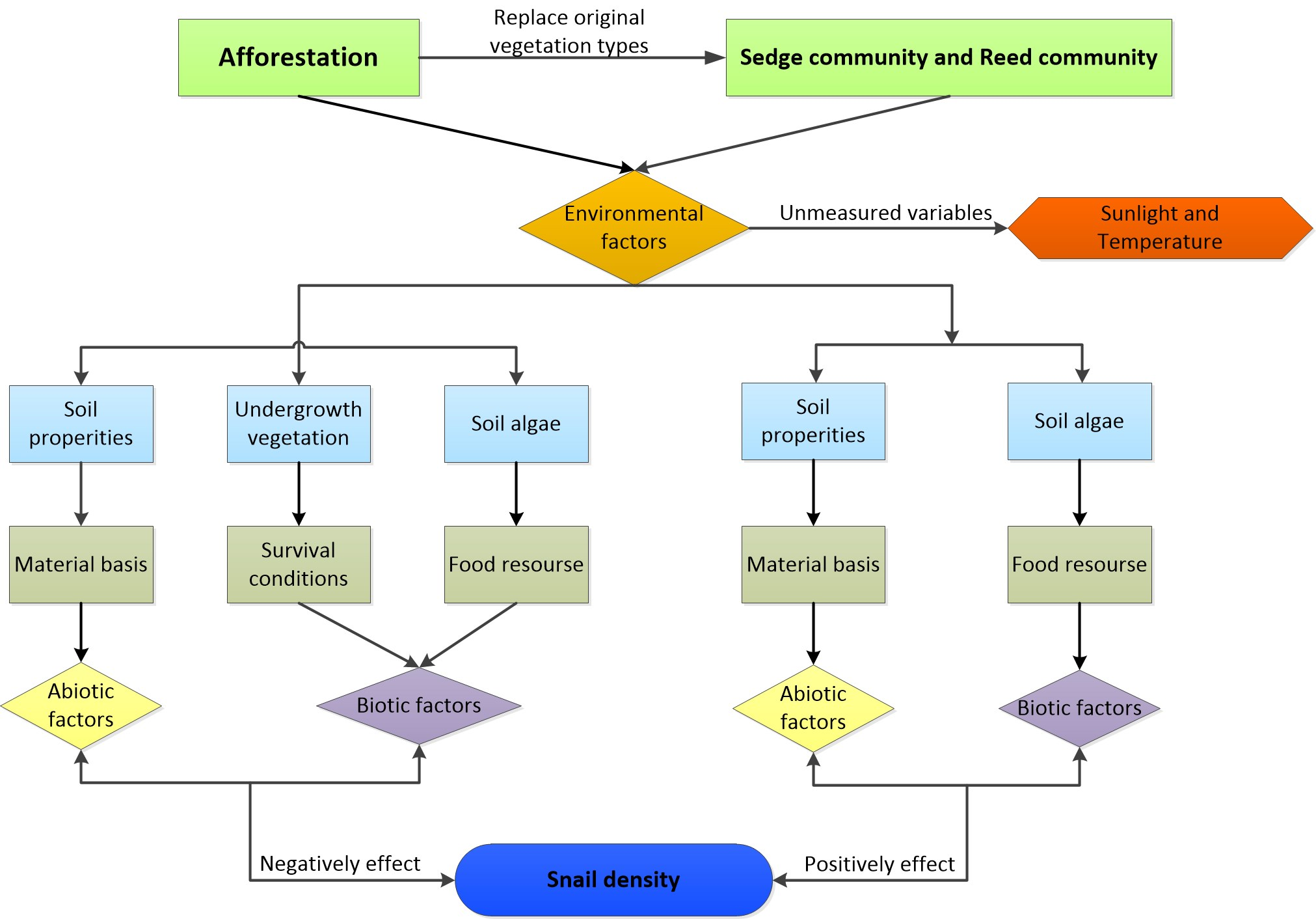

Supplement: S4 Fig — (TIF) [file pntd.0009100.s008.tif]

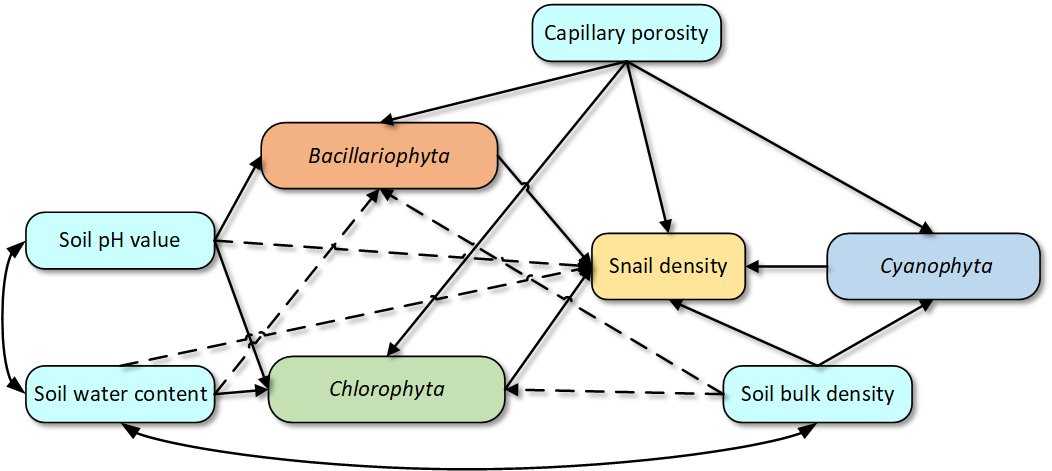

Supplement: S5 Fig — (TIF) [file pntd.0009100.s009.tif]
